# Effect of Low Temperature on Growth and Ultra-Structure of Staphylococcus *spp*

**DOI:** 10.1371/journal.pone.0029031

**Published:** 2012-01-24

**Authors:** Laura A. Onyango, R. Hugh Dunstan, Johan Gottfries, Christof von Eiff, Timothy K. Roberts

**Affiliations:** 1 Environmental and Pathogenic Microbiology Laboratory, School of Environmental and Life Sciences, University of Newcastle, Newcastle, New South Wales, Australia; 2 Department of Chemistry, Gothenburg University, Gothenburg, Sweden; 3 Institute of Medical Microbiology, University of Münster, Münster, Germany; Indian Institute of Science, India

## Abstract

The effect of temperature fluctuation is an important factor in bacterial growth especially for pathogens such as the staphylococci that have to remain viable during potentially harsh and prolonged transfer conditions between hosts. The aim of this study was to investigate the response of *S. aureus*, *S. epidermidis*, and *S. lugdunensis* when exposed to low temperature (4°C) for prolonged periods, and how this factor affected their subsequent growth, colony morphology, cellular ultra-structure, and amino acid composition in the non-cytoplasmic hydrolysate fraction. Clinical isolates were grown under optimal conditions and then subjected to 4°C conditions for a period of 8 wks. Cold-stressed and reference control samples were assessed under transmission electron microscopy (TEM) to identify potential ultra-structural changes. To determine changes in amino acid composition, cells were fractured to remove the lipid and cytoplasmic components and the remaining structural components were hydrolysed. Amino acid profiles for the hydrolysis fraction were then analysed for changes by using principal component analysis (PCA). Exposure of the three *staphylococci* to prolonged low temperature stress resulted in the formation of increasing proportions of small colony variant (SCV) phenotypes. TEM revealed that SCV cells had significantly thicker and more diffuse cell-walls than their corresponding WT samples for both *S. aureus* and *S. epidermidis*, but the changes were not significant for *S. lugdunensis*. Substantial species-specific alterations in the amino acid composition of the structural hydrolysate fraction were also observed in the cold-treated cells. The data indicated that the staphylococci responded over prolonged periods of cold-stress treatment by transforming into SCV populations. The observed ultra-structural and amino acid changes were proposed to represent response mechanisms for staphylococcal survival amidst hostile conditions, thus maintaining the viability of the species until favourable conditions arise again.

## Introduction

While staphylococci belong to the common flora of the skin and mucous membranes, they can quickly become opportunistic pathogens when the host's immune system is breached to cause an array of diseases. Various strains of *S. aureus* have become noted for their resistance to several antimicrobial agents and together with a range of coagulase-negative staphylococci (CNS), including *S. epidermidis* and *S. lugdunensis*, these bacteria account for a significant proportion of nosocomial infections [1], [2], [3].

Exposures of staphylococci to antibiotics have been shown to be one cause for the formation of small-colony variants (SCVs). These variants represent sub-populations of bacteria that exhibit atypical growth features from those seen in their wild-type (WT) counterparts. As the name suggests, these variants are characterized by mostly non-haemolytic and non-pigmented tiny colonies, about 1/10 the size of their WT counterparts [4]. Interest in SCVs forms emerged when they were associated with persistent clinical infections [5], [6]. In humans, SCVs have been associated with chronic persistent infections of the skeletal system, the heart and lungs, and other organ sites, also when indwelling medical devices were used [7]. Treatment of such infections has become a challenge since SCVs were shown to be less susceptible to several antibiotics, and additionally these phenotypes persist better intracellularly within host cells [8]. Studies including animal models found that many virulent factors expressed by the WT phenotypes in causing disease were either not expressed or remained minimal in the SCV populations [9], [10], [11]. Instead, SCVs up-regulated mechanisms that would support their attachment and uptake into host cells, as well as instigating metabolic strategies that would promote their survival once internalized without having to use cytotoxic measures [12]. Reports of multiple auxotrophism [5], [13], [14], [15] suggested that certain metabolic pathways were inactivated in SCVs. Further molecular studies have provided evidence that the clinical SCVs were different phenotypes compared to their WT parents but were capable of reversion to the WT form [16], [17] suggesting that the phenotypic change involved significant alterations in metabolic homeostasis in the SCV.

Staphylococci require an ability to survive on inanimate objects through the transition processes from one host to another. They must be able to adapt to rapidly changing environmental conditions and be ready to reactivate metabolism and virulence factors when opportunities arise. Temperatures of 4°C are often utilised for storage of food products, solutions and biological materials since the majority of pathogens are considered mesophilic and therefore do not to grow well at this temperature [18]. Staphylococci are able to grow over a wide temperature range (6.5–46°C) although their optimal range is 30–37°C and it has been suggested that they can survive at extremes of <6.5°C and >46°C for limited periods of time [18]. The ability of staphylococci to rapidly adapt to fluctuating low and high temperatures is particularly crucial for pathogenic strains of staphylococci since there are instances where these bacteria have to remain viable outside of a host [19].

The bacterial cell-wall and its associated proteins represent the interface between the environment and the cytoplasm. It acts as a structural barrier against toxic chemicals, protects the cell against fluctuating environmental conditions and plays an important role in infection and pathogenecity [20]. Several studies have examined the cell-wall structure of antibiotic-resistant *S. aureus* strains and the results showed that cell-wall thickening was a common characteristic produced only when the bacterium was grown in the presence of antibiotics [21]. In these studies, the proposed function of a thickened cell-wall was to bind and sequester vancomycin further away from its target site. The hypothesis was thus proposed that bacterial cells exposed to a range of stressors could adapt the composition of their cell-wall and associated proteins to facilitate protection against changing environmental conditions. To potentially measure these altered composition, an analysis of the non-cytoplasmic fraction and specifically of amino acid changes would be investigated.

In the present study, the investigation set out to determine whether exposure to low temperature at 4°C for 8 weeks would instigate the formation of SCV phenotypes as a survival mechanism for clinical isolates of *S. aureus*, *S. epidermidis*, and *S. lugdunensis*. Furthermore, the rates of changes in SCV numbers as proportions of the viable bacterial populations were determined over the 8-week exposure time and any associated changes in cell-wall morphology and composition were assessed by TEM and GC-MS respectively. It was hypothesised that the exposure to cold stress would result in increased cell-wall thickness and altered biochemical amino acid composition.

## Materials and Methods

### Bacterial samples

*S. aureus*, *S. epidermidis*, and *S. lugdunensis* were isolates derived from an earlier investigation [22] and maintained as culture stock within the laboratory. The isolates had been appropriately stored and routinely sub-cultured to maintain viability. Identity checks were performed regularly by PCR and standard API® Staph biochemistry.

### Bacterial growth

Overnight broth cultures of *S. aureus*, *S. epidermidis*, and *S. lugdunensis* were used to generate fresh liquid cultures (100 mL grown in 250 mL flasks) which were grown to mid-exponential phase at 37°C, 120 rpm and thereafter incubated at 4°C for 8 weeks. Each week, n = 9 samples were obtained from these cold-stressed liquid cultures and diluted 1∶10 before plating 5 µl aliquots of each sample in triplicate onto Columbia horse blood agar (HBA, Oxoid). Inoculated plates were incubated for 24 hrs at 37°C and thereafter examined. Regular purity checks were performed on the stored broth cultures by PCR-based assays to ensure no cross contamination had occurred during sampling. Colonies growing from the sub-cultured stressed broths were analysed for culture purity. This was performed by use of the API® Staph test (bioMérieux) and by PCR-based assays of the 16SrRNA gene using the method of Brown *et al.*, [23] and the results verified through the NCBI BLAST database (http://www.ncbi.nlm.nih.gov/BLAST/).

### Colony characterization

Colonies were physically assessed and categorized into two groups based on size, haemolytic activity and pigmentation. Colonies were characterized as SCVs if they presented as pinpoint colonies (<1 mm in diameter) with decreased haemolytic activity and pigmentation 24–48 hrs post-incubation as described in literature [17], [24], [25] All other colonies were characterized as wild-type (WT).

### Reversion

SCVs generated from cold-temperature treatment were tested for reversion by sub-culturing individual colonies (n = 9) onto HBA plates overnight under optimal conditions, and the rate of reversion recorded as the percentage of WT colonies in the overall population.

### Specimen preparation for TEM

The TEM sample preparation procedure used was combined from methods of Glauert [26], and Dykstra and Reuss [27]. N = 9 colonies of both WT and SCV colonies grown on HBA were fixed in 2.5% glutaraldehyde. Secondary fixation was done in 1% osmium tetroxide solution. Dehydration was performed in a graded water- ethanol series (v/v) made to the following concentrations: 10%, 30%, 50%, 70%, 90% and 100%. Infiltration was performed in LR white resin made up in ethanol to the following concentrations: 10%, 30%, 50%, 70%, 90% and 100% (v/v). Ultra-thin sections were cut stained and examined under a transmission electron microscope set at an acceleration voltage of 80 kV and images taken at 40–100, 000X.

### Sample preparation for amino acids studies

Following the 8 week exposure period, cells were harvested and extracted for cell-wall and membrane components according to the methods of Hanaki *et al.* [28] and de Jonge *et al.* [29]. Cells were disrupted by boiling with glass beads for ½ hr. The suspension was centrifuged at 3000 g for 5 min and the supernatant discarded. The cell pellets containing the cell-wall and membrane fragments were then lyophilized. The membrane lipid components of the lyophilized samples were extracted by adding chloroform: methanol in a ratio of 2∶1 v/v for a final volume of 3 mL. The samples were mixed and then centrifuged for 5–10 min at 3500 rpm and 4°C. The lower chloroform phases were aspirated to 8 mL extraction tubes. This procedure was repeated twice more with the addition of chloroform alone and the resulting chloroform phases combined. The remnant methanol suspensions were transferred into 8 mL derivatisation tubes and centrifuged for 5–10 min. These pellets were lyophilized to remove the methanol before hydrolyzing in acid. The dried methanol pellet was hydrolysed by adding 200 µl of 6 M HCL to the sample and the suspension incubated for 6 hrs at 100°C. Thereafter, the samples were cooled and freeze-dried.

### Amino acid analyses of hydrolysed cell wall/protein extract

Dried hydrolyzed samples were prepared for analysis by use of the Ez∶Faast™ kit (Phenomenex® EZ∶faast™) and separated for analysis by gas chromatography (GC) which is suitable for the detection of over 40 amino acids and related derivatives. The procedure involves a solid phase extraction by ion exchange chromatography, derivatisation to form propyl esters of amino acids and a final liquid/liquid extraction step prior to analysis by GC. The lyophilised hydrolyzed samples were re-suspended in 500 µl of milliQ water and extracted following the 8-step procedure as directed by the manufacturer using norvaline as the internal standard. Analysis of the EZ∶Faast™ derivatised samples was performed on a Hewlett Packard HP 6890 series GC system fitted with a flame ionisation detector, and ZB-PAAC-MS column (10 m×0.25 mm id), supplied by Phenomenex® Inc. The instrument method comprised split injection (ratio 15∶1), with injector temperature 250°C and a column flow rate of 0.5 mL/min. Injection volume was set at 2.5 µl for all samples.

### Statistical analysis

Experiments were conducted three times (each in triplicate, n = 9) to ensure reproducibility of results. Principal Component Analysis (PCA) was conducted using SIMCA-p+ (12.0, Umetrics Sweden), [30]. All data were pre-treated by log transformation, mean centering and unit variance scaling prior to PCA generation. Optimal PCA model complexity was decided according to the Cross Validation (CV) procedure [31].. The CV included seven leave out data modelling rounds while at the same time all data were left out once, as implemented in SIMCA-P+. Outlier data scanning was undertaken by critical orthogonal distance to model, and by Hotelling's T^2^ within model dimensions. Neither of the methods indicated any spurious data. Loadings confidence intervals were estimated by Jack-knifing using the CV results.

## Results

**Figure 1:**
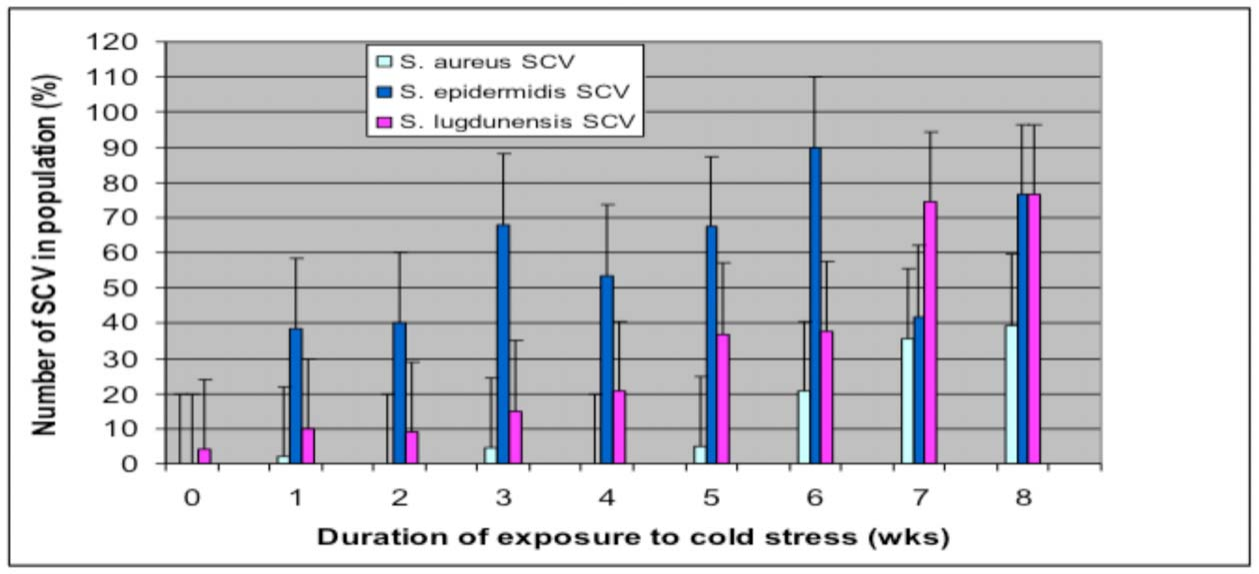
The change in the percentage compositions of SCV colonies of populations of *S. aureus S. epidermidis* and *S. lugdunensis* cultures exposed to temperature stress at 4°C for a period of 8 wks (n = 9).

Broth cultures of *S. aureus S. epidermidis* and *S. lugdunensis* exposed for extended periods to temperatures of 4°C after initial growth at 37°C yielded a range of colony variants on subculture onto HBA plates with variations in size, pigmentation, and haemolytic activity. The ability to form small colony variants (SCV) in response to 4°C stress was observed in all three staphylococcal species, although their frequencies within the populations differed between species, and their abundances were associated with the duration of stress. During the initial stages of stress (1–2 wks), the most abundant colony type seen in culture for each species was the WT colony. SCVs were also present at this time although they represented <40% of the population (Figure 1). However, with prolonged incubation under temperature stress, there was a shift in population dynamics with SCV colonies representing >50% of the population after 3 wks for *S. epidermidis* (R^2^ = 0.51, p<0.05) and 7 wks for *S. lugdunensis* (R^2^ = 0.97, p<0.05). Cultures of *S. aureus* did not yield significant SCV numbers until after five weeks of incubation (R^2^ = 0.78, p<0.05).

**Figure 2:**
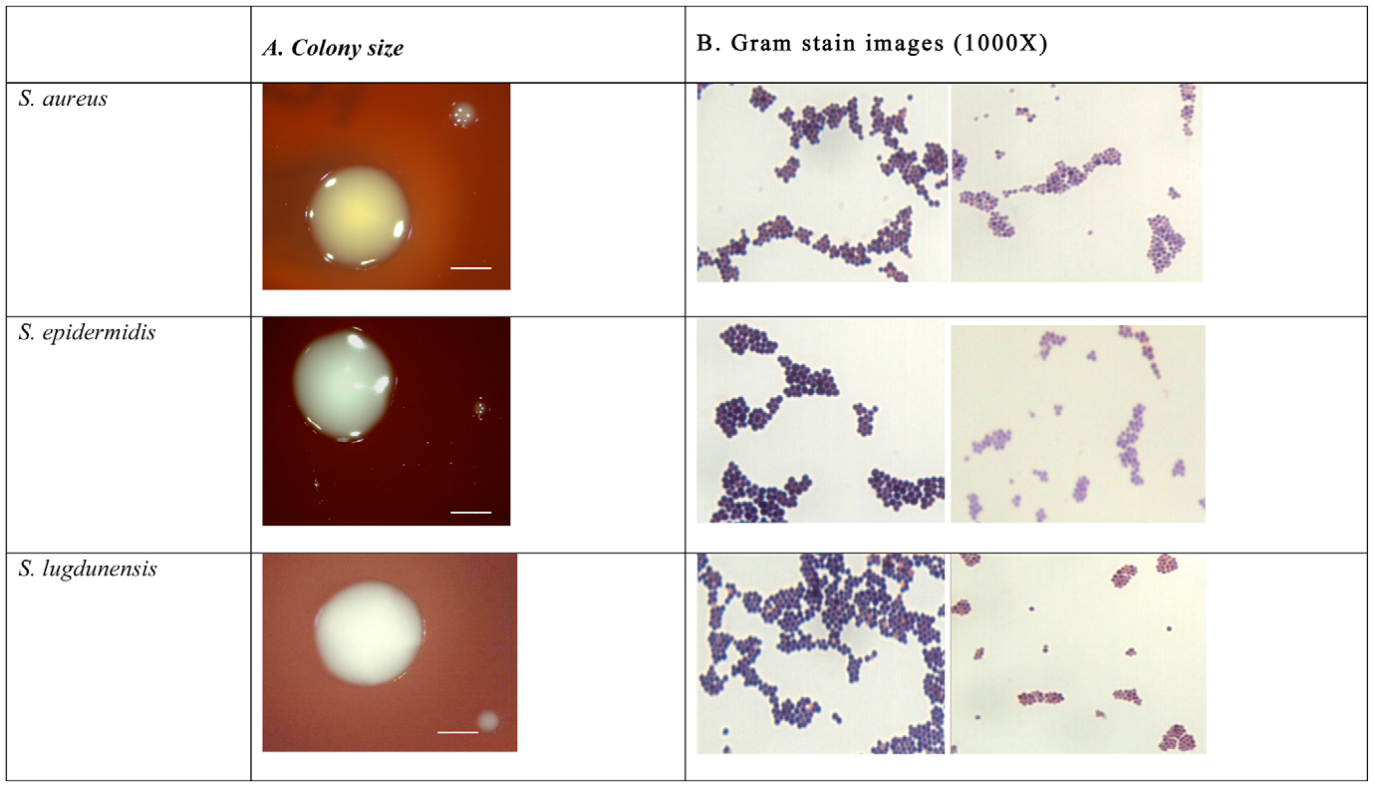
Morphological variations in size, pigmentation, haemolysis and Gram stain between WT and their corresponding SCVs in *S. aureus*, *S. epidermidis* and *S. lugdunensis*. Column (A) shows differences in size and pigmentation between WT colonies (left) being larger and more pigmented than their SCVs (right) which are minute with diminished pigmentation (scale bar represents 1 mm). Column (B) shows the differences in response to Gram staining with WT cells (left) staining significantly darker than their corresponding SCVs (right).

Temperature-induced SCVs were <1 mm in colony size and showed reduced pigmentation with slight and variable ranges of haemolysis on HBA (Figure 2). Comparisons of the Gram stained cells are also summarised in Figure 2 showing a noticeable difference in the intensities of the stains between each colony type for all 3 species examined. A simplistic colour scale was used to determine the intensity of the dye and a scoring scale of 1–5 used, with 5 being the darkest intensity and 1 the lightest intensity. WT colony cells consistently stained a dark purple and had a mean value ±SD of 4.7±1.0 (n = 20) while SCV cells always stained significantly lighter with a mean value of 2.1±1.0 (n = 20).

### Species identification

To ascertain that the observed mixed population of colonies were not contaminants, WT and suspected SCVs of all three species were assessed by API® Staph readings and by PCR of the 16S rRNA gene. PCR of all tested SCV colonies confirmed their identity as consistent with the original WT stock. The API® Staph results for the WT colonies tested positive for their corresponding stock species, but those of the SCVs were inconclusive which was consistent with the proposed alterations in metabolic homeostasis reported in the literature. To further assess the identity of the SCVs, these colonies were shown to revert to WT colonies when sub-cultured onto HBA plates and incubated at 37°C. The SCV's reverted after 2 sub-cultures under for cells stored for 4 weeks or less at 4°C, and up to 3 sub-cultures for reversion to be observed for cultures stored from 5–8 weeks. These revertants formed from sub-culture in this manner were subsequently confirmed as similar to the parent strains by both API® Staph and PCR.

### Electron microscopy

**Figure 3:**
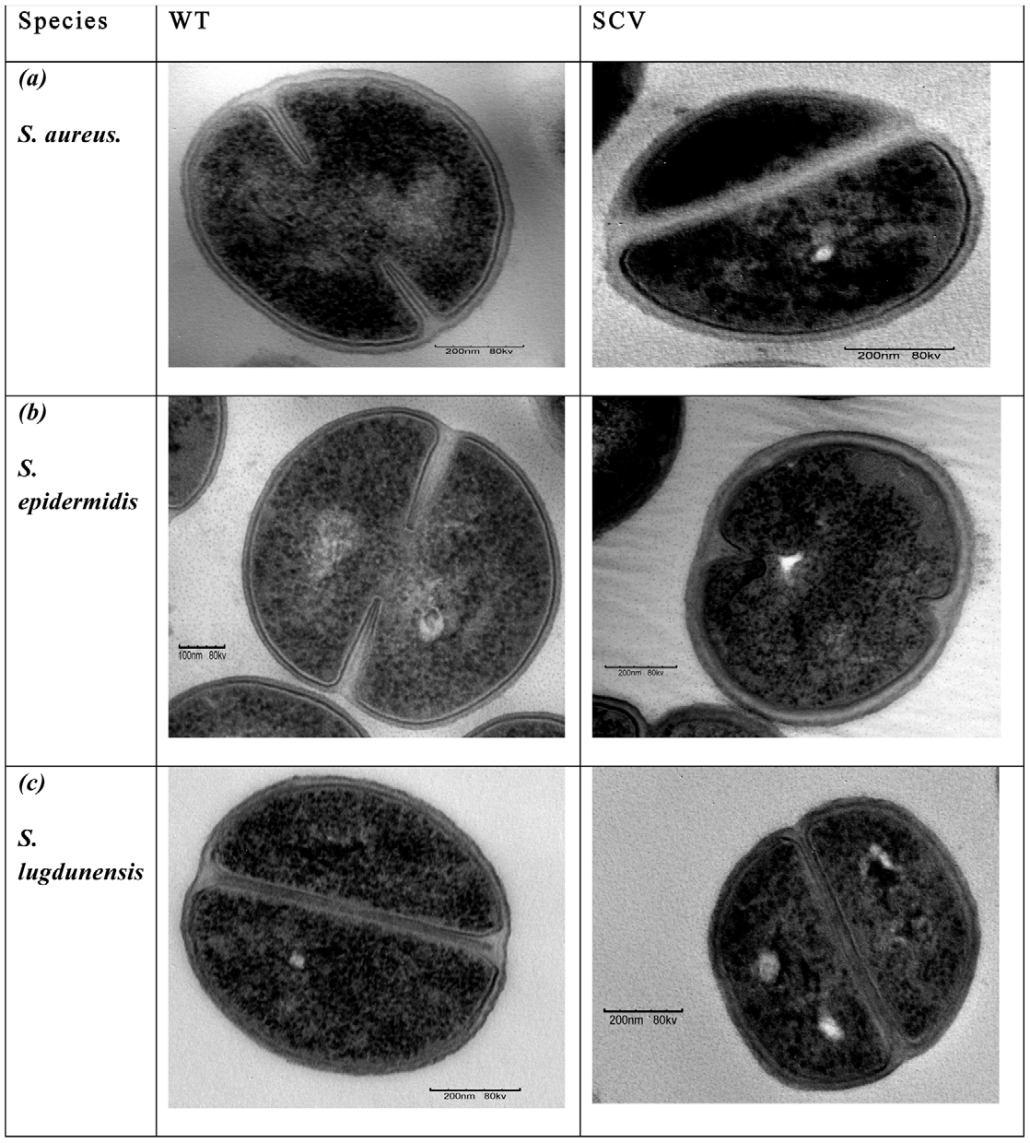
TEM images of (a) *S. aureus*, *(b) S. epidermidis* and (c) *S. lugdunensis* WT and SCV cells showing their respective ultra-structural characteristics. WT cells had clearly defined cell-walls in comparison to SCVs which had thicker, more diffuse cell-walls following exposure to stress (4°C).

Fixed WT and SCV colonies (n = 9 each) were sectioned and examined using the TEM. Results revealed irregularities in the appearance of cytoplasmic components, septa formations, cell symmetry, and cell-wall properties from SCV cells compared with their parental WT cells. Micrographs of WT cells of each species showed well defined septa formation occurring primarily through the middle of the cell, dividing the cell into two symmetrical daughter cells (Figure 3). Conversely, SCV cells exhibited more diffuse septa and predominantly more “asymmetrical” cell divisions where one daughter cell appeared to be substantially smaller than the other as shown in Figure 3(a) for the *S aureus* SCV. An appraisal of symmetrical versus asymmetrical cell-divisions in *n* = 100 cells from each colony type from all three species was performed and summarised in Table 1, which confirmed that SCV populations had a significantly greater proportion of their cells with apparent “asymmetrical” cell divisions compared with their corresponding WT cells (p<0.01).

**Table 1:** Analysis of the incidence of apparent “symmetrical” *vs.* “asymmetrical” cell divisions in WT *vs.* SCV cells from TEM cell preparations of *S. aureus*, *S. epidermidis* and *S. lugdunensis*.

Colony type	Symmetrical cell division	Asymmetrical cell division
*S. aureus* WT	62.2%	37.8%
*S. aureus* SCV	19.3%	80.7%
*S. epidermidis* WT	70.6%	29.4%
*S. epidermidis* SCV	35.0%	65.0%
*S. lugdunensis* WT	66.9%	33.1%
*S. lugdunensis* SCV	28.5%	71.5%

Further TEM analyses showed that SCV cells appeared to have more diffuse cell-walls that also appeared to be thicker than their parental strains. To ascertain whether there were indeed significant differences, measurements of wall-thickness were performed for n = 300 cells per colony type for each species. For each cell, measures were taken from three separate regions and the average of these values recorded as the wall-thickness of that particular cell. For each replicate prepared, at least ten micrographs were recorded and 10 fields of view were examined per micrograph to record the various data (diffuse and thickened cell-walls, and asymmetrical septa formation) and ascertain that the ultra-structural changes were not artefacts. Analyses of these data revealed significant differences in wall thickness between WT and SCV colonies of *S. aureus* and *S. epidermidis*. The mean (±SD) cell-wall thickness for *S. aureus* SCV (24±7 nm) was significantly thicker than their corresponding WT cells (17±3 nm). *S. epidermidis* SCV cells were also significantly thicker (33±15 nm) when compared with their WT cells (27±5 nm). There was no significant difference in cell-wall thickness between *S. lugdunensis* WT cells (24±5 nm) and their corresponding SCV cells (24±6 nm).

### Principal Component Analyses

Due to the changes in morphology observed in the TEM micrographs of SCV cells following exposure to 4°C, it was hypothesised that there would be concomitant changes in composition of cell-wall and cell wall associated proteins. The cell-wall fractions derived from SCV and WT cells of *S. aureus*, *S. epidermidis* and *S. lugdunensis* were thus digested and hydrolysed for determinations of amino acid composition by gas chromatography. The amino acid composition data were collated from the 3 staphylococci isolates grown as reference controls in cultures at 37°C or subjected to prolonged cold stress at 4°C (n = 9 for each combination). The full data matrix was subjected to PCA rendering a 3 Principal Components (PC) solution, according to cross validation (CV), with explained variances of 82% R^2^X and 72% by CV (Q^2^X). However, the third PC contributed less than 5% to cumulative CV and was therefore disregarded.

**Figure 4:**
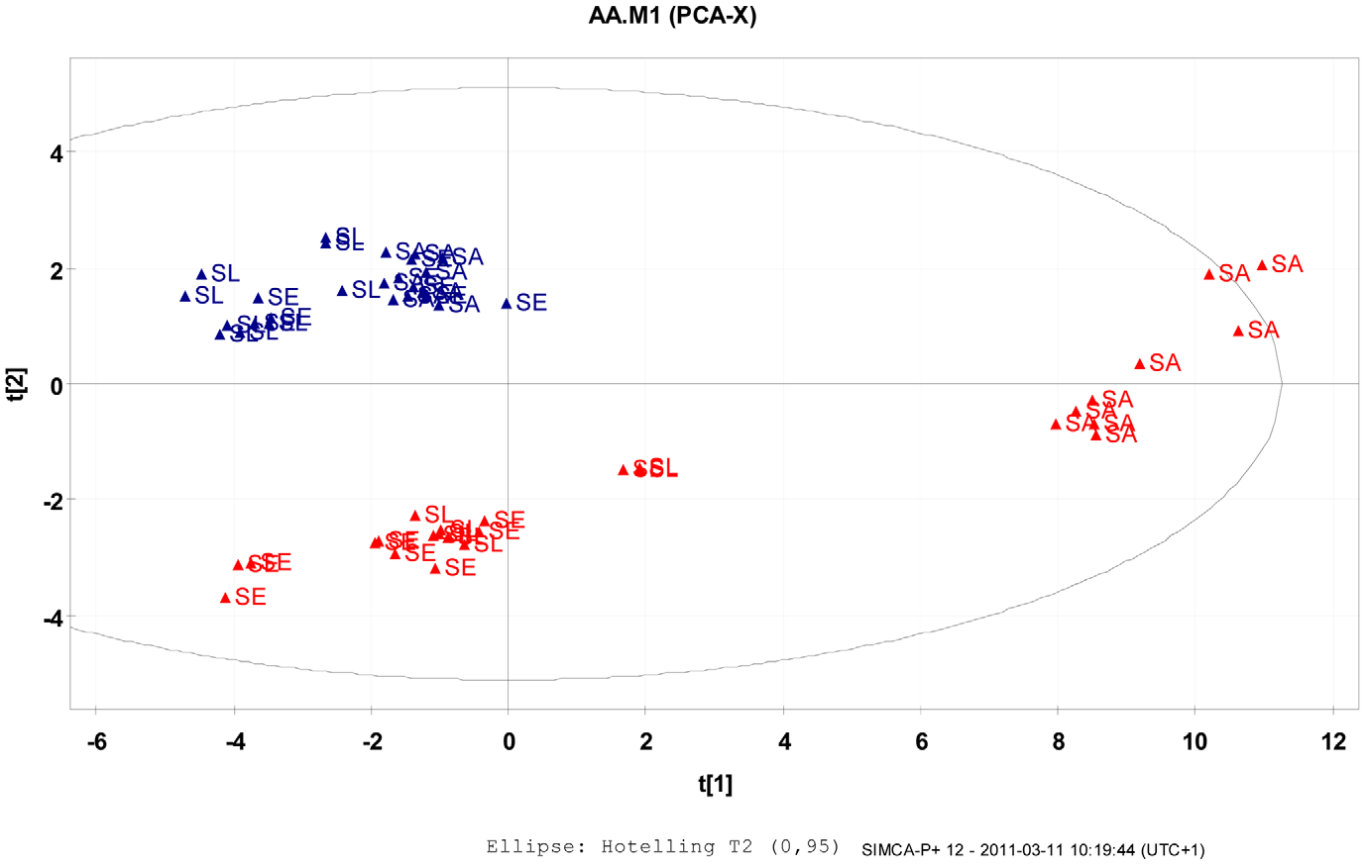
Principal Component Analysis (PCA) scores (t1 versus t2) scatter plotted from staphylococcal cell-wall amino acid profile data. The staphylococci cultures were grown under ideal conditions at 37°C representing the reference control samples (Cont) or subjected to prolonged exposure to 4°C for 8 weeks (TE) before sampling and amino acid analyses. Three different strains, *S. aureus* (SA), *S. epidermidis* (SE) and *S. lugdunensis* (SL) were investigated in replicates (n = 9) for each strain for responses to the temperature conditions.

Inspection of the PCA scores revealed 3 clusters whereby the samples from staphylococci, independent of strain grown at 37°C, were all positioned to form a reference group modeled close to the origo (Figure 4). The cold treated *S. aureus* samples formed a second cluster which was modeled with generally high t1-scores for all amino acids but close to zero t2 scores. This orientation indicated that all amino acids were more abundant in all cold treated *S. aureus* samples as compared with the reference samples. In contrast, the cold treated *S. epidermidis* and *S. lugdunensis* samples which overlapped each other, comprised close to zero scores in the first PC but low *t*2 scores compared to the reference samples. The data indicated that all 3 species had similar amino acid composition profiles in the cell-wall when grown under optimal conditions at 37°C, i.e. the reference samples. However, when the cells were subjected to the prolonged cold stress at 4°C, all 3 strains responded by substantially altering composition of amino acids in their cell-walls and associated proteins. However, *S. aureus* displayed a very unique and characteristic response which was clearly different to that evoked in *S. epidermidis* and *S. lugdunensis*.

**Figure 5:**
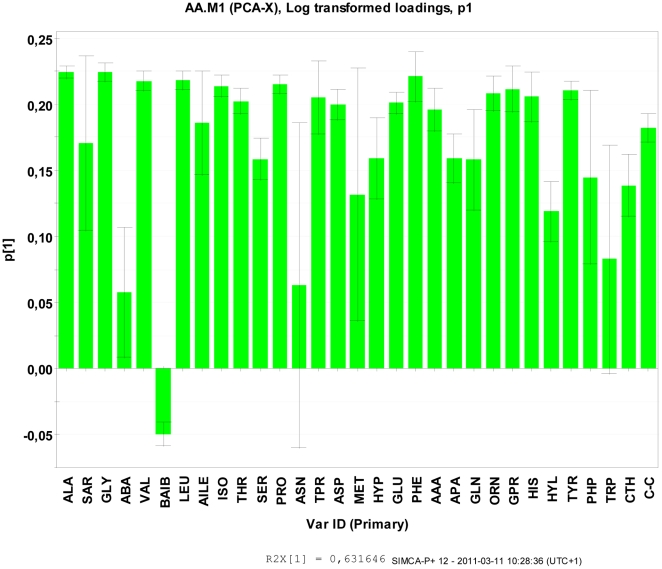
The p1 scores for amino acids from the cell wall extracts of *S. aureus* following prolonged exposure at 4°C for 8 weeks compared with corresponding cultures grown under ideal conditions at 37°C representing the reference control samples. *S. aureus* responded to the cold stress by generally increasing amino acid composition in this fraction relative to the control, i.e. reference samples from all three strains.

The positioning of all the cold treated *S. aureus* samples to the right of the PCA scatter-plot was due to high t1 scores (as shown in Figure 4), which resulted from increased amino acid levels in all *S. aureus* samples (see PC1, i.e. the x-axis in Figure 4). The individual amino acid level correlation patterns to the PC-components were derived by the loadings. These were defined as the correlation between the individual PCs to the individual amino acid abundance in the included samples. For the first PC in the present study all correlations were positive for the *S. aureus* samples compared to reference, given by the generally positive correlation structure by *p*1 loadings. This full correlation pattern between *S. aureus* cold treated and reference samples were in accordance to the loadings displayed in Figure 5.

**Figure 6:**
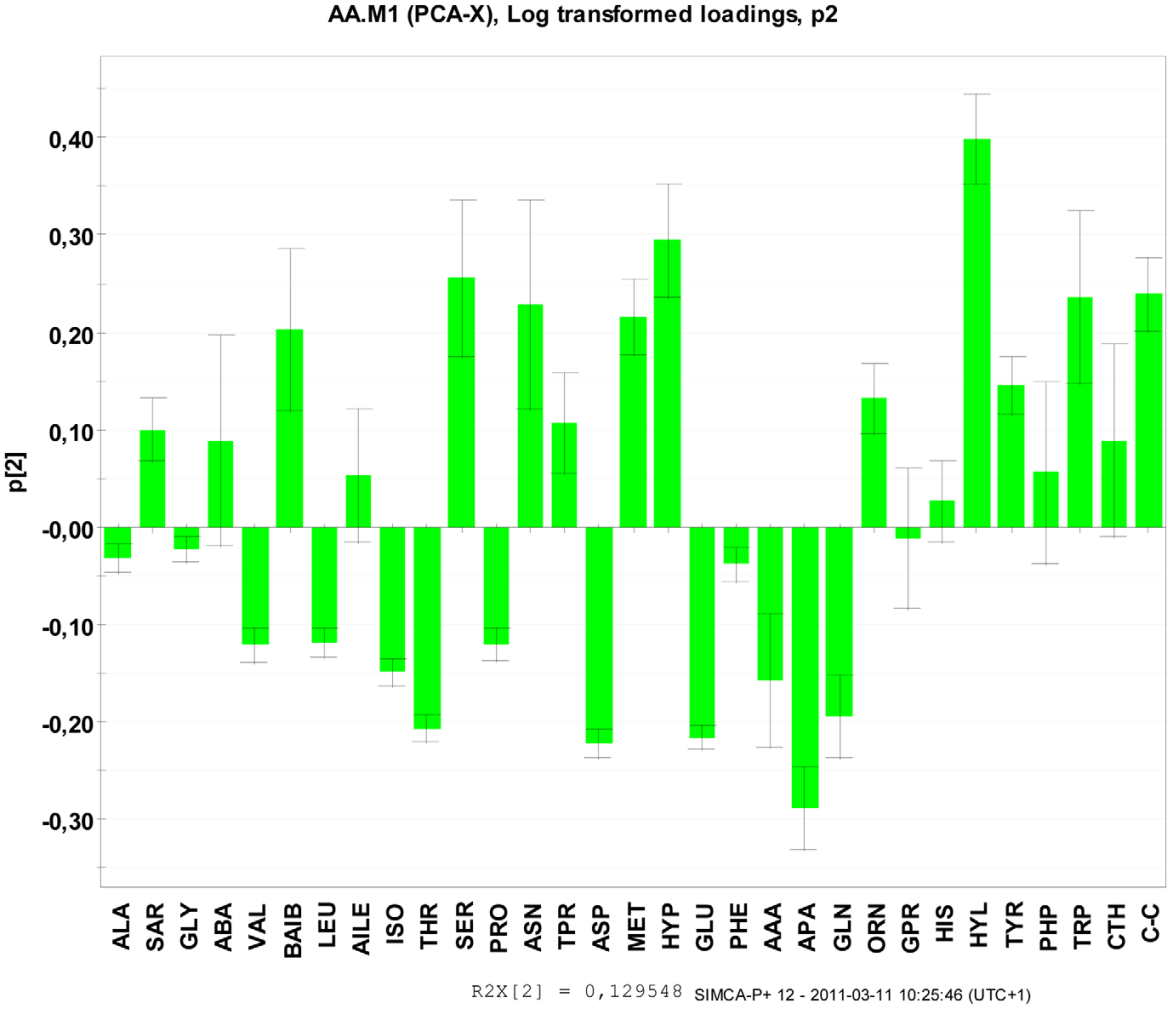
The p2 scores for amino acids from the cell wall extracts of *S. epidermidis* and *S. lugdunensis* following prolonged exposure at 4°C for 8 weeks compared with corresponding cultures grown under ideal conditions at 37°C representing the reference control samples. *S. epidermidis* and *S. lugdunensis* responded to the cold stress by substantially altering amino acid profiles relative to the respective controls. It should be emphasized that the PC2 were inversely correlated to cold treatment such that negative loadings, e.g. - 0,20 for GLN, indicated increased amino acid levels in the cold treated *S. epidermidis* and *S. lugdunensis* samples, and vice versa.

The amino acid changes characterizing the responses from cold treatment of *S. epidermidis* and *S. lugdunensis* were orthogonal to the *t*1 response since the individual samples varied mainly along the PC2 direction (Figure 4). In addition, the PCA correlation direction was inverse compared to PC1 as indicated by the positioning of cold treated *S. epidermidis* and *lugdunensis* samples by lower *t*2 scores as compared with the reference samples. In practice it meant that negative loadings in PC2 indicated increased amino acid levels in the cold treated samples from *S. epidermidis* and *lugdunensis* as compared to the reference samples, e.g. APA and GLN were generally higher and SER and ASN were lower in the cold treated *S. epidermidis* and *lugdunensis* samples as compared to reference samples. These responses involved significant alterations in amino acid profiles in comparison with their respective control cultures, with increases in e.g. SER, ASP and MET and decreases in e.g. THR, GLU and GLN (see all loadings for PC2 in Figure 6).

## Discussion

When staphylococcal cultures were exposed to the 4°C temperature challenge, *S. epidermidis* and *S. lugdunensis* cultures generated SCVs more readily than cultures of *S. aureus*. Ultimately, all three species formed SCV colonies, but there was never 100% conversion during the 8-week period of exposure to 4°C. The time dependent increases in the proportions of SCV's in the sampled populations suggested a process of adaptation, where the increasing predominance of SCVs represented natural selection of the SCV's with enhanced capacity for survival under adverse conditions [32]. Phenotypic switching has been noted in *S. aureus* as an effective bacterial strategy against host immune response and the successful establishment of chronic infection [33]. This phenomenon is of evolutionary significance as it offers a selective advantage for bacterial survival in the face of adverse environmental conditions. In this study, it was proposed that this mechanism would be entirely appropriate for survival periods between potential hosts for these commensal (opportunistic pathogens) in contrast with the spore forming bacteria such as bacilli, which may require survival for extended periods of time under more extreme conditions of water deprivation, exposure to UV radiation and toxic chemicals. Although formation of SCVs occurred in *S. aureus*, *S. epidermidis* and *S. lugdunensis* following exposure to 4°C, it was noted that the SCV's became more frequent in coagulase-negative staphylococci (CNS) cultures at a faster rate than in *S. aureus*. Colony variation has previously been observed in CNS in response to antibiotic treatment [34], but it was suggested that perhaps this feature may have been under-reported since the altered morphological characteristics were misinterpreted as contaminations rather than representing phenotypic diversity [34].

The use of Gram staining was employed as a routine procedure as part of the species identification process. It was quickly noticed that the SCV cells were consistently stained lighter than their corresponding WT cells, indicating possible irregularities between their cell-wall structures which is the basis of this staining technique. Assessment of multiple samples using a simple visual analogue scale revealed significant differences in stain intensity providing further evidence that alterations in cell-wall and associated protein composition may be associated with the formation of SCV cells in response to cold stress. A study performed by Sieradzki and Tomasz [21] involving a vancomycin resistant mutant of *S. aureus* reported that this mutant had copius amounts of peptidoglycan and extracellular debris that had the appearance of cell-wall material. The build up of cell-wall material was thought to sequester vancomycin from the medium thereby preventing it from reaching its target and damaging the cell. It is plausible that the thickened cell-wall observed in SCV samples of this current study could have prevented some of the crystal violet dye from penetrating within the wall thereby resulting in the lighter Gram's stains observed much in the same way that Sieradzki and Tomasz reported that thicker cell-walls sequestered and bound antibiotic thereby preventing its penetration into the cell. The hypothesis was proposed that the thicker cell-walls of *S. aureus* and *S. epidermidis* SCV cells combined with alterations in amino acid composition in all 3 species limited the uptake of the Gram stain.

TEM micrographs of *S. aureus* and *S. epidermidis* SCV cells showed diffuse cell-walls that were significantly thicker in these staphylococcal species whereas no significant differences were observed between WT and SCV cells for *S. lugdunensis*. Cell-wall thickening has been noted as a resistance mechanism employed by *S. aureus* against clinically-important antibiotic penetration [35], [36]. In our study, SCVs were generated by exposure to temperature stress with similar results suggesting that cell-wall thickening may be a generic response of bacterial cells exposed to stress. A thicker cell-wall may act as a protective mechanism for the cell against extracellular challenges that may otherwise compromise the viability of the bacterium. In antibiotic-resistant CNS, the diffuse nature of the cell-wall was identified as cell-wall bound slime that was thought to enhance adherence and colonization by these species thereby making them highly successful in causing device-related pathogenesis [37]. Similar responses by *S. aureus*, *S. epidermidis* and *S. lugdunensis* instigated by exposure to colder conditions would also convey appropriate advantages for adhesion to and survival on fomites for transfer between hosts. It is clear that *S. lugdunensis* elicited a different cellular response to the cold treatment compared with *S. aureus* and *S. epidermidis* by having no measurable alterations in cell-wall thickness. The responses of *S. aureus* and *S. epidermidis* were consistent with the hypothesis that the exposure to cold could result in thickening of cell-walls as observed in antibiotic treated cells [35], but this is not necessarily the case for all staphylococci.

Further analyses of TEM micrographs showed that SCV samples had more diffuse septa in their dividing cells. These samples also had a significantly higher proportion of apparent “asymmetrical” cell divisions in *S. aureus S. epidermidis* and *S. lugdunensis* with a smaller daughter cell observed in 81, 65 and 72% of SCV samples compared with only 38, 29 and 33% seen in WT samples, respectively. Kahl and associates have also [15] documented impaired septa formation in their investigation of clinical *S. aureus* SCVs, and proposed that it may have led to impaired cell separation. Sianglum *et al* [38] noted the same result when *S. aureus* was treated with a novel antibiotic and concluded that the antibiotic interrupted processes in cell division. Sanyal and Greenwood [39] observed this characteristic in *S. epidermidis* cultures treated with the antibiotic teicoplanin. TEM micrographs of their samples showed cells having undergone cell division into two daughter cells but the septa were formed more towards one pole of the cell rather than right through the cell's middle, similar to what was observed in this current study. They did not address the possible reasons for this characteristic.The ultra-structural changes observed in *S. aureus S. epidermidis* and *S. lugdunensis* in response to prolonged exposure to 4°C were consistent with the hypothesis that a survival phenotype was produced in the stressed bacterial populations which increased in frequency with prolonged exposure. It was proposed that such a response would coincide with alterations in biochemical composition of the cell-wall structure to optimise survival for the cell. The analyses of digested wall and protein extracts from cells harvested after growth in ideal conditions (WT) compared with those from cells harvested following a subsequent 8 weeks of storage at 4°C revealed substantial alterations in amino acid composition.

Inspection of the PCA scores indicated an orthogonal difference in amino acid composition of the cell-wall and associated proteins between low temperature-treated *S. aureus* on one hand, versus *S. epidermidis* and *S. lugdunensis* on the other hand. The comparison was made using the WT amino acid measures for all three staphylococcal species and straight forward interpretation applies since little variation in amino acid levels was observed within the WT independent of strain. The mathematical interpretation of the apparent orthogonal clustering of the *S. aureus* versus *S. epidermidis* and S. *lugdunensis* in the PC1 versus PC2, entails a complete difference in physiological response between the two clusters. The increases in amino acid concentrations observed in *S. aureus* were interpreted to represent a proportional increase in cell-wall associated proteins with associated qualitative differences, since all measured amino acids were elevated to varying degrees. In contrast, *S. epidermidis* and *lugdunensis* appeared to display substantial qualitative changes in amino acid composition that would be consistent with qualitative changes in protein inclusion. It was further concluded that the proportions of cell-wall associated proteins in *S. epidermidis* and *S. lugdunensis* were similar to their respective control WTs.

The data suggest that all three species adapt by changing the amino acid composition profiles of the cell-wall and associated structural proteins, which implies altered protein composition. Further conclusions regarding a possibly unique adaption strategy for *S. aureus*, within the different Staphylococcus strains upon environmental challenge would need additional empirical data. However, the present findings would be in line with the clinical observations of severe pathogenicity observed in *S. aureus* infections as compared to other staphylococcal species which could be explained by improved survival by swift and radical adaption abilities [40]. Future studies would include trying to identify the proteins alterations that may occur in response to environmental stresses. An enhanced understanding of the cell-wall associated protein responses to environmental stimuli and stresses may provide new insights for developing antimicrobial strategies.

In conclusion, the results of this study credibly showed that exposure of *S. aureus*, *S. epidermidis* and *S. lugdunensis* to cold temperature instigated morphological, ultra-structural and biochemical changes in cell-wall composition which governs their phenotypic outcomes. The changes associated with the SCV phenotype were transitory rather than genetic, evidenced by the ability to revert to a WT phenotype upon sub-culture without stress. Rapid switching between two phenotypes allows for bacterial flexibility that serves as an advantage when swift responses to environmental fluctuations are required.
